# Ursolic Acid Derivatives as Potential Agents Against *Acanthamoeba* Spp.

**DOI:** 10.3390/pathogens8030130

**Published:** 2019-08-23

**Authors:** Ines Sifaoui, Rubén L. Rodríguez-Expósito, María Reyes-Batlle, Aitor Rizo-Liendo, José E. Piñero, Isabel L. Bazzocchi, Jacob Lorenzo-Morales, Ignacio A. Jiménez

**Affiliations:** 1 Instituto Universitario de Enfermedades Tropicalesy Salud Pública de Canarias, Universidad de La Laguna, Avda Francisco Sanchez s/n, Campus de Anchieta, 38271 La Laguna Tenerife, Islas Canarias, Espana; 2 Laboratoire Matériaux-Molécules et Applications, University of Carthage, La Marsa Tunisia, Tunisia; 3 Instituto Universitario de Bio-Orgánica Antonio González, Departamento de Química Orgánica, Universidad de La Laguna, Avenida Astrofísico Francisco Sánchez 2, 38206 La Laguna, Tenerife, Islas Canarias, Espana

**Keywords:** ursolic acid derivatives, *Acanthamoeba*, chemotherapy, programmed cell death

## Abstract

The current chemotherapy of *Acanthamoeba* keratitis relies on few drugs with low potential and limited efficacy, for all this there is an urgent need to identify new classes of anti-*Acanthamoeba* agents. In this regard, natural products play an important role in overcoming the current need and medicinal chemistry of natural products represents an attractive approach for the discovery and development of new agents. Ursolic acid, a natural pentacyclic triterpenoid compound, possesses a broad spectrum of activities including anti-*Acanthamoeba*. Herein, we report on the development by chemical transformation of an ursolic acid-based series of seven compounds (2–8), one of them reported for the first time. The structure-activity relationship (SAR) analysis of their anti-*Acanthamoeba* activity revealed that acylation/ether formation or oxidation enhances their biological profile, suggesting that the hydrophobic moiety contributes to activity, presumably by increasing the affinity and/or cell membrane permeability. These ursolic acid derivatives highlight the potential of this source as a good base for the development of novel therapeutic agents against *Acanthamoeba* infections.

## 1. Introduction

Ursolic acid (UA) is a natural pentacyclic triterpenoid compound derived from the berries, leaves, flowers, and fruits of medicinal herbs, such as *Rosemarinus officinalis*, *Oldenlandia diffusa*, and *Radix actinidiae*. It has been reported to possess various effects such as antiviral [1], antibacterial [2], anti-inflammatory [3], and anticancer activities [4]. Its antitumor effects have been investigated in vitro and in vivo and as inducing tumor differentiation, inhibiting the proliferation of cancer cells, inducing apoptosis, as well as promoting the autophagy of cancer cells. Furthermore, UA could inhibit the proliferation of cervical cancer lines, including HeLa, CaSki, and SiHa, furthermore, inducing apoptosis in HeLa cells [5]. Few reports have described the action mode of UA toward the *Leishmania* parasite. In this field, UA isolated from *Petiveria alliaceae* induced programmed cell death (PCD) in the promastigote form of *Leishmania amazonensis* and early alterations in the mitochondria physiology of the parasite [6].

*Acanthamoeba* is one of the pathogen genera of free-living amoeba, widely distributed in the environment, it has been isolated from different habitats namely soil, water, air, etc. *Acanthamoeba* exists as two stages in their life cycle: A motile, trophic, and replicating trophozoite and a resistant cyst stage. It could directly infect animal and humans causing several diseases like *Acanthamoeba* keratitis (AK) and granulomatous amebic encephalitis (GAE) or by acting as a 'Trojan horse' for infective bacteria namely *Legionella pneumophila* [7]. Recently, *Acanthamoeba* keratitis cases are constantly increasing due to the higher number of contact lens wearers [8]. The existence of a resistance stage; cyst in the *Acanthamoeba* genus is the main concern in the development of effective therapeutic agents. Currently, the first line treatments against *Acanthamoeba* keratitis are inefficient, due to the appearance of resistance strains, and long-term use of eye drops are highly toxic for the patients [9,10]. Moreover, lately and due to the lack of efficacy of the present treatment, the research for new effective drugs against this parasite has grown exponentially.

In a previous report, we have pointed out the amoebicidal activity of UA. In fact, we observed that this triterpenic acid could induce apoptosis in *Acanthamoeba castellanii* Neff by decreasing the mitochondrial membrane potential and the ATP levels produced in cells [11]. 

Due to its low solubility in water, the use of ursolic acid in the pharmaceutics industry is still limited [12]. To overcome these problems as well as to improve its selectivity effects towards parasites, several derivatives from UA have been synthetized and proved to exhibit higher biological activity than the origin compound [12]. In this context, the aim of this study was to evaluate the amoebicidal and the toxic effects of ursolic acid derivatives and to elucidate the in vitro effect of the most effective molecules.

## 2. Results

### 2.1. Chemistry

**Figure 1:**
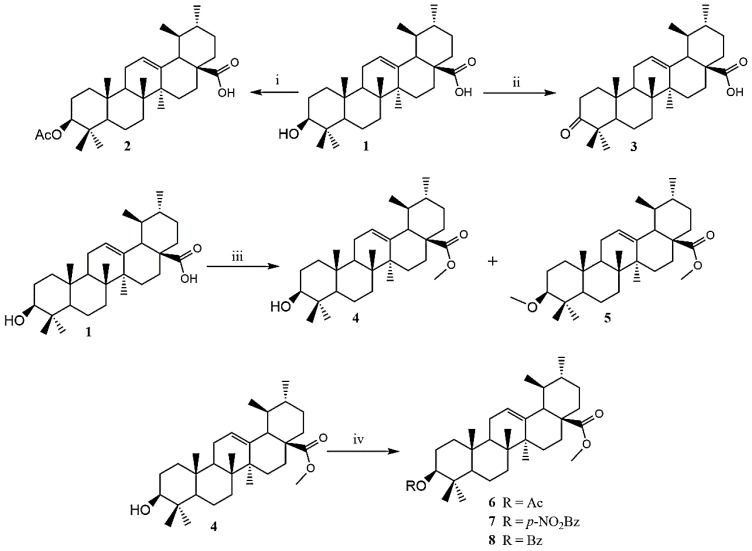
Synthesis of ursolic acid analogues 2–8. Reagents and conditions: (i) Ac_2_O, Et_3_N, CH_2_Cl_2_; (ii) PCC, acetone; (iii) CH_3_SO_4_, K_2_CO_3_, acetone; and (iv) Ac_2_O/p-NO_2_BzCl/BzCl, Et_3_N, CH_2_Cl_2_.

The synthesis of seven analogues was carried out by alkylation, acylation, or oxidation of the hydroxyl group of the C-3 position or carboxylic acid of the C-28 position of UA ‘1’ as presented in Figure 1. Among the former synthetic derivatives, one out of seven was reported for the first time: Derivative 7. The structure of the new compound was elucidated by high-resolution mass spectrometry and NMR analysis. The known derivatives 2–6 and 8 were elucidated by comparison of their spectral data with those reported in the literature.

### 2.2. Biological Study

The corresponding in vitro amoebicidal activities results are summarized in Table 1. The 50% inhibitory concentration (IC_50_) was chosen as the appropriate and comparable data to give as previously described [13]. The chemical modifications of UA enhanced the anti-*Acanthamoeba* activities with an IC_50_ ranging from 40.0 for 1 to 21.4 for the 7 and from 15.3 for 1 to 6.7 for 7, respectively for *A*. Neff and *A. griffini*. 

**Figure 2:**
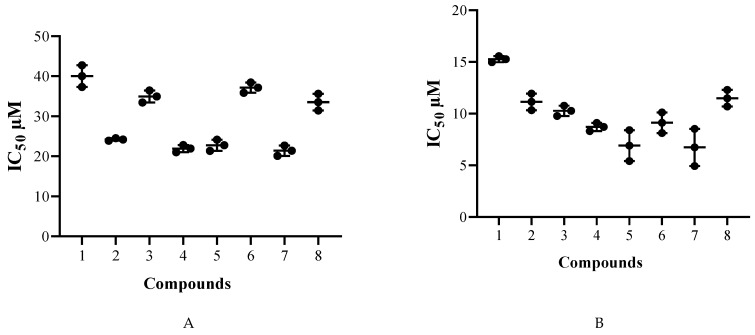
Distribution of the IC_50_ of the trophocidal activities for *Acanthamoeba castellanii* Neff (**A**) and *Acanthamoeba griffini* (**B**).

The analysis of variance by a two-factor ANOVA, illustrated that the biological activity was strain dependent with *p* < 0.0001. In fact, the *A. griffini* was more sensitive to the derivatives than *A.* Neff. The anti-trophozoite effect of the molecule was statistically significant with *p* < 0.0001. According to the Figure 2A, for *Acanthamoeba castellanii* Neff, all the derivatives except 6 were statistically more effective than the UA. 

As for the *Acanthamoeba griffini* species, all the derivatives were statistically more effective than UA as could be observed in the Figure 2B.

**Figure 3:**
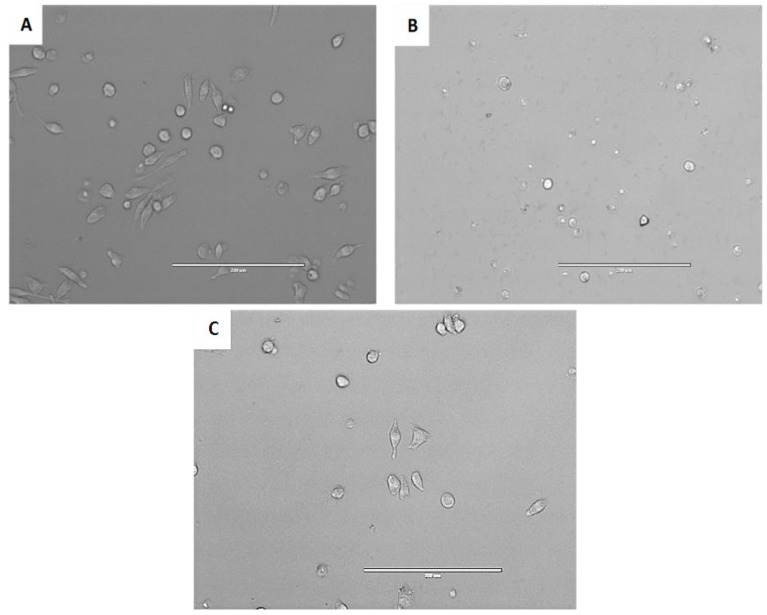
Effect of an ursolic acid derivative at 50 µM in murine macrophages (J774A.1) observed by inverted microscopy (20×) . Negative control (**A**), 1 (**B**) and 5 (**C**).

Considering the cytotoxicity towards the murine macrophage, if the inhibition could not reach 50% at the highest concentration, then >100 µM was given. Our data showed that the UA was the most toxic followed by 4 and 2. Meanwhile, the other six derivatives possessed low toxicity with a CC_50_ higher than 100 µM. In fact, as could be observed in Figure 3, at 50 µM 5 did not affect the morphology of the murine macrophages, compared to 1.

**Figure 4:**
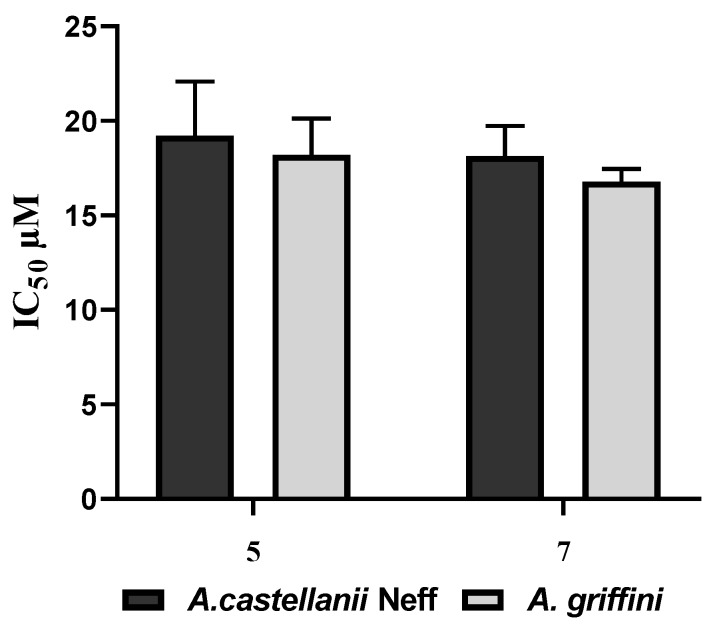
Histogram plot of the cysticidal effects of both derivatives 5 and 7 showing the viability of *Acanthamoeba* spp. cysts.

Based on their activity against *Acanthamoeba* and the toxicity towards the macrophages, compounds 5 and 7 were chosen for further studies. Both derivatives were observed to cause a dose-dependent cysticidal effect. As shown in the histogram of Figure 4 For both strains no significant difference was observed in the effect of both molecules.

**Figure 5:**
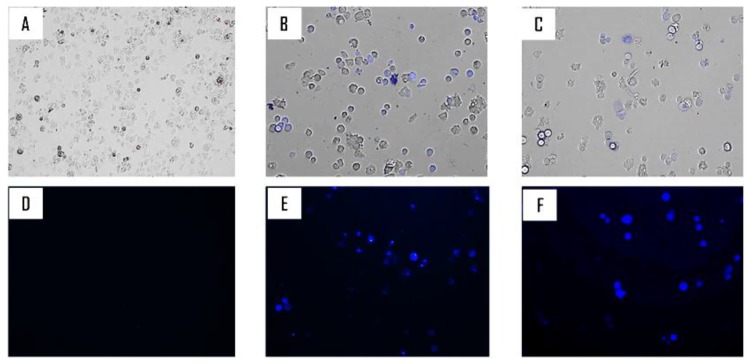
*Acanthamoeba castellanii* Neff trophozoite incubated with IC_90_ of 5 (**B, E**) and 7 (**C, F**) for 24 h. Negative control (**A, D**). Hoechst stain is different in control cells, where uniformly faint-blue nuclei are observed, and in treated cells, where the nuclei are bright blue. Images (20×) are showing chromatin condensation (blue) in treated cells. (**A** to **C**). Overlay images: (**D** to **F**) Hoechst channel (Magnification of 20× ).

Derivatives 5 and 7 treated cells stained positive in the double-stain assay. When double staining was performed, the tested drug at a concentration of IC_90_ 86 µM and 75 µM respectively for 5 and 7could induced chromatin condensation proved by the bright-blue nuclei stain (Figure 5), although the Propidium Iodide (PI) did not stain (data not shown).

**Figure 6:**
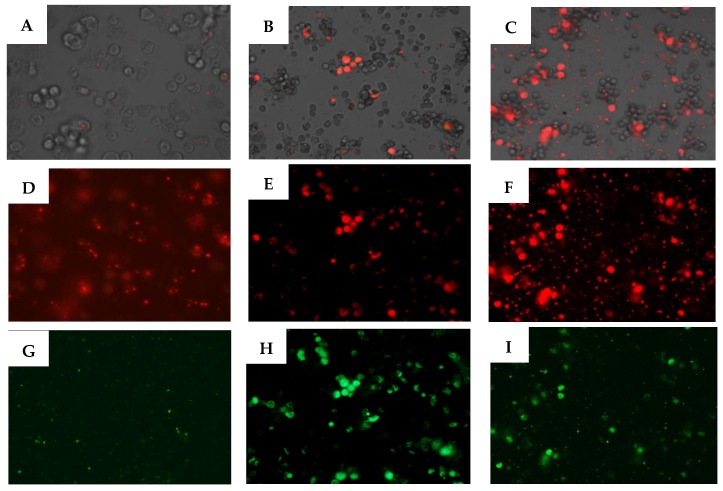
The effect of IC_90_ of 5 (**B, E, H**) and IC_90_ of 8 (**C, F, I**) on the mitochondrial potential, JC-1 dye accumulates in the mitochondria of healthy cells as aggregates (red fluorescence) in cells treated with the urolic acids derivatives for 24 h, due to the collapse of the mitochondrial potential, the JC-1 dye remained in the cytoplasm in its monomeric form, green fluorescence. Images are representative of the population of treated amoeba (20×). (**A** to **C**) Overlay images, (**D** to **F**) JC-1 aggregates channel, and (**G** to **I**) JC-1 monomers channel.

Compounds 5 and 7 induced mitochondrial damages. As illustrated in the Figure 6, both derivatives could depolarize the mitochondrial membrane potential by inhibition of JC-1 agglomeration and emission of green fluorescence. 

**Figure 7:**
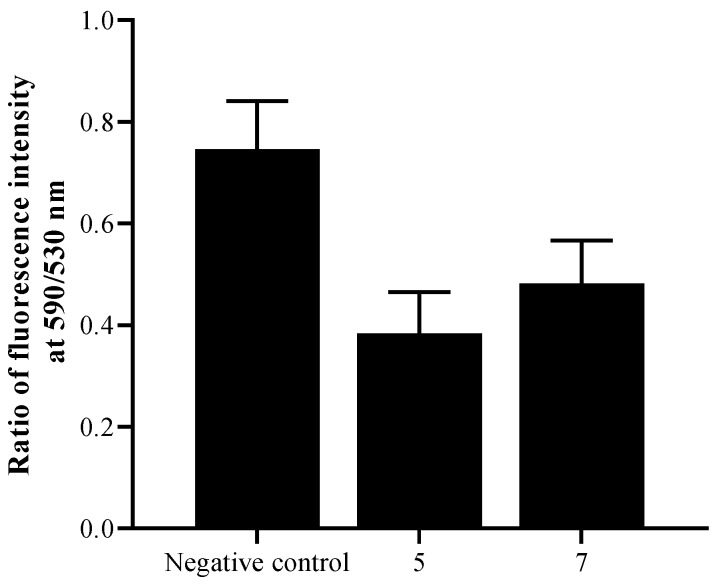
Mitochondrial membrane potential (Δψ_m_) showing a change in the ratio of fluorescence intensity at 590/530 nm after the 24 hours of treatment with both IC_90_ of 5 and 7.

Histograms of the fluorescence (Figure 7) demonstrated that the treatment with 5 and 7 decreased the *Acanthamoeba castellanii* Neff mitochondrial membrane potential (Δ*Ψm*) by 49.0% and 35.0% when treated with IC_90_, respectively. 

**Figure 8:**
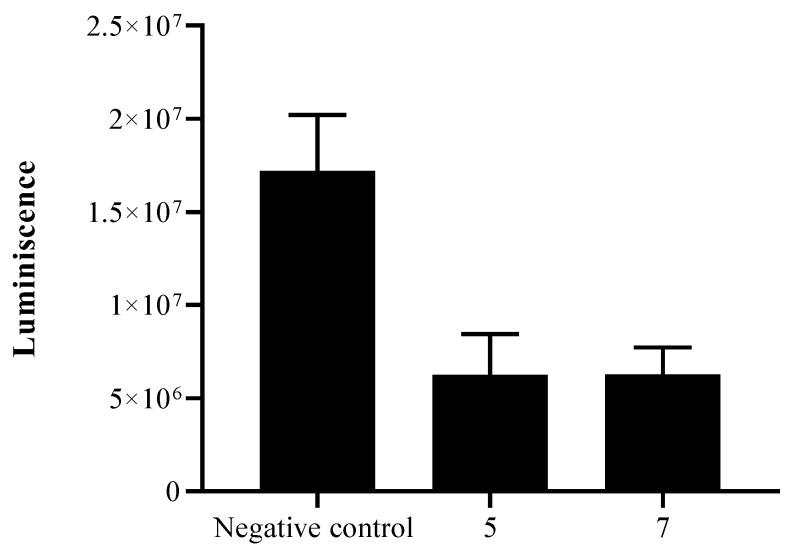
The effect of both molecules on the ATP content, using a CellTiter-Glo® Luminescent Cell Viability Assay. Results represent the percentage relative to the negative control.

The mitochondrial damage was also checked by measuring the ATP level generated in 24 h. We found out that the treated cells with the IC_90_ produced half of the ATP produced by the untreated cells (Figure 8).

**Figure 9:**
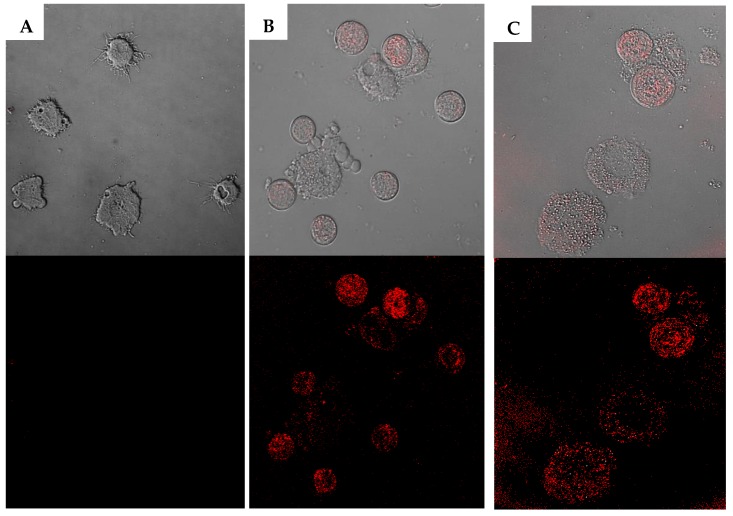
Increased levels of ROS in *Acanthamoeba* Neff incubated with compounds 5 (**B**) and 7 (**C**) for 24 h. Negative control (**A**): Cells exposed to CellROX® Deep Red in the absence of triterpenic acids. Images (63×) are representative of the cell population observed in the performed experiments. Cells were observed in a Leica TSC SPE- confocal microscope.

Derivatives 5 and 7 increased reactive oxygen species (ROS) levels in *Acanthamoeba*. Treated amoebae with the IC_90_ for 24 hours generated higher level of ROS than the negative control demonstrated by the red fluorescence emitted (Figure 9).

## 3. Discussion

Organic chemistry plays an important role in the pharmaceutical industry and drug discovery process [14]. Even though, the natural products serve as a source of raw material for innovative drug research, a chemical modification remains imperative to enhance the efficacy/safety profile of a new drug [15,16,17,18]. Latterly, many attempts on structural modifications of UA have been made to improve its efficacy and specificity against cancer cells [19], HIV virus [20], and *Plasmodium falciparum* [21,22], among others.

The hit compound (UA, 1) and their derivatives (2–8) were evaluated in vitro for their growth inhibition capacity against the trophozoite stage of *Acanthamoeba* spp: *A. castellanii* Neff and *A. griffini*, and against murine macrophages, wherein the latter used to calculate the selectivity index. All derivatives made in this study demonstrated in general higher anti-*Acanthamoeba* activity than the origin product, with lower toxicity towards murine macrophages.

Moreover, it is noteworthy that analogues 2, 4, 5, and 7 were the favorable trend for optimal trophocidal activity against *A. castellanii* Neff, with an activity two-fold higher than the original product. Taking into consideration the promising results obtained for UA derivatives on *A. castellanii*, this series of compounds was evaluated against *A. griffini*. The results indicated that all analogues exhibited a higher effect than the hit compound, UA. Moreover, compounds 5 and 7 displayed a growth inhibition of *A. griffini* two-fold higher than the UA and with an IC_50_ of 6.9 and 6.7 µM, respectively.

After confirming that the analogues showed potent activity against both strains, the cytotoxicity against murine macrophages was tested to determine the selectivity index as shown in Table 1. According to Suffnes et al. (1999) the selectivity index (SI) is considerately good when its value is higher than 2 [23]. Among the evaluated compounds, five of them showed a selectivity index higher than 10 for *A. griffini* than the murine cell line, including the most active analogues on this strain, compound 5 and 7. Furthermore, five compounds had a SI higher than the reference drug chlorhexidine.

The influence of the substitution pattern of the uran-12-ene skeleton on the amoebicidal activity was examined, revealing the following trends on the preliminary structure-activity relationship. As shown in Table 1, compound 1 that contained two oxygenated positions, a hydroxyl group at C-3, and a carboxylic acid at C-28, showed lower potency than those analogues (2–8). These results indicated that a substituent at C-3 or C-28 on the core skeleton is relevant for the activity. Moreover, the substituent at C-3 is crucial for the inhibition of the parasite, with the presence of a hydroxyl group being unfavorable with respect to an ester or ketone (1 versus 2 and 3). In addition, esterification of carboxylic acid has a favorable effect on the activity, as revealed by comparison of potency of the hit compounds 1 with its methyl ester derivative 4. Our results were accordingly similar to the anti-tumor activity of UA reported by several authors. In general, the acetylation of the C-3 of UA revealed to be beneficial for obtaining high anti-tumor activity [24,25]. Our findings support that lipophilicity plays an important role in the trophocidal capacity of these series of compounds especially on the cytotoxicity profile.

In the last decades, programmed cell death (PCD) or apoptosis-like has been described in unicellular protists, including the *Plasmodium*, *Trypanosoma*, and *Leishmania* genera, and *Acanthamoeba* [11,26]. As originally defined in multicellular organisms, apoptosis refers to certain morphological modifications that occur during genetically controlled cell death. This process shows a common event in both multicellular and unicellular including chromatin condensation, loss of mitochondrial membrane potential, nuclear DNA fragmentation, cell shrinkage, and the formation of apoptotic bodies [26,27]. Hoechst 33342 is a selective DNA fluorochrome used to investigate the chromatin condensation [28]. Our results indicated that 5 and 7 induced chromatin condensation in treated cells, and resulted in the induction of apoptotic cell death. In the same idea, Chen et al. (2019) have reported that ursolic acid DNA condensation and fragmentation (ladder) resulted in the induction of apoptotic cell death in NCI-H292 cells [29].

Depolarization of the mitochondrial membrane potential is a substantial evidence of apoptosis induction. The (ΔΨm) was measured by spectrofluorometric analysis using the mitochondrial membrane potential sensitive cationic dye JC-1 [30]. In healthy cells, the dye accumulates in the mitochondria as aggregates and gives a red fluorescence signal. In apoptotic cells, the mitochondrial potential collapses, and the JC-1 does not accumulate in the mitochondria and remains in the cytoplasm giving a green fluoresce signal. Both molecules 5 and 7 induced mitochondrial malfunction. Several reports, confirmed the mitochondria transmembrane potential collapse in different cancer cells treated with ursolic acid [31]. In our previous work, we have proved the effect of UA activating the PCD in *Acanthamoeba* by affecting the mitochondrial membrane potential [11]. Additionally, other authors showed the apoptotic effect of this triterpenic acid in cancer cell lines. Li et al. (2014) observed that ursolic acid was able to inhibit the proliferation of HeLa cells by inducing apoptosis via the mitochondrial intrinsic pathway [32]. Furthermore, the effect of ursolic acid (UA) on the proliferation of the human breast cancer cell line, MDA-MB-231 was investigated by Kim et al. (2011). They found that UA inhibited the proliferation of the cancer cells through both a mitochondrial death pathway and extrinsic death receptor dependent pathway [33].

The mitochondrial membrane potential is crucial for ATP generation in the respiratory chain [34]. Furthermore, affecting the mitochondria function will provoke a decrease in intracellular ATP produced [34,35]. Herein, the ATP level was affected as a result of the mitochondria collapse.

Another aspect of the mitochondrial malfunction is the generation of oxidative stress in the cells. In fact, the oxidative stress is known as imbalance of the ROS production over antioxidant defenses [36]. Our results show a higher production of ROS in the treated cells comparing to the negative control, which will confirm again the mitochondria damage. Isopropyl 3β-hudroxyurs-12-en-28-oat a derivative from ursolic acid to induce apoptosis in NTUB1 cells (human bladder cancer cells) via the excessive production of ROS [37]. 

In this study it was demonstrated that both compounds 5 and 7 possessed a selective and interesting in vitro activity against *Acanthamoeba* strains. Additionally, the solubility of both analogues was higher than the origin compound 1 (ursolic acid), which would ease their application in drug formulation as eye drops to treat AK. To enhance the amoebicidal activity of the actual treatment a formulation based on one of the actual derivatives and a currently used therapeutic agent such as chlorhexidine or biguanide could be a good option. Nevertheless, further studies using in vivo models, which would allow us to obtain data such as tissue penetration and drug half-life among others should be developed in the near future.

## 4. Materials and Methods 

### 4.1. Molecules/Chemicals

Ursolic acid was purchased from Extrasynthese (Cymit quimica, Barcelona, Spain). The compounds tested in this study are shown in Figure 1.

The known derivatives were prepared according to the literature: 2 [24,38], 3 [24,38], 4 [24,38], 5 [38,39], 6 [40,41], and 8 [42,43]. 

Derivative 7 was prepared as follows: A solution of compound 4 (6 mg), two drops of dry trimethylamine, and 8 mg of *p*-nitrobenzoyl chloride in dry CH_2_Cl_2_ (1 mL) were stirred under argon atmosphere at room temperature. The progress of the reaction was monitored by Thin Layer Chromatography (TLC) using hexane/ethyl acetate (9:1). After the mixture was concentrated to dryness under reduced pressure, the residue was purified by preparative-TLC using hexane/ethyl acetate (9:1) to afford the corresponding compound 7 (5.4 mg).

3β-(p-nitrobenzoyloxy)-urs-12-en-28-oic acid methyl ester (**7**).

Light white lacquer; [α] ^20^_D_ = +33.3 (c 0.24, CHCl_3_); for 1^H^ NMR (CDCl_3_, 600 MHz), δ 0.80 (3H, s, H-24), 0.90 (3H, d, *J* = 6.4 Hz, H-29), 0.95 (1H, m, H-5), 0.97 (3H, s, H-23), 0.98 (3H, d, *J* = 6.4 Hz, H-30), 1.03 (3H, s, H-25), 1.05 (3H, s, H-26), 1.11 (1H, m, H-15), 1.12 (3H, s, H-27), 1.19 (1H, m, H-1), 1.33 (1H, m, H-21), 1.37 (1H, m, H-7), 1.44 (1H, m, H-6), 1.52 (1H, m, H-21), 1.55 (1H, m, H-7), 1.59 (1H, m, H-6), 1.60 (1H, m, H-19), 1.61 (1H, m, H-9), 1.62 (1H, m, H-22), 1.70 (1H, m, H-22), 1.71 (1H, m, H-16), 1.75 (1H, m, H-1), 1.81 (2H, m, H-2), 1.82 (1H, m, H-15), 1.95 (H, m, H-20), 1.96 (2H, m, H-11), 2.04 (1H, m, H-16), 2.67 (1H, d, *J* = 11.6 Hz, H-18), 4.81 (1H, dd, *J* = 6.4, 10.1 Hz, H-3), 5.28 (1H, t, *J* = 3.4 Hz, H-12), 3.64 (3H, s, OCH_3_), *p*-NO_2_Bz [8.22 (2H, d, *J* = 8.9 Hz), 8.31 (2H, d, J = 8.9 Hz)]. ^13^C NMR (CDCl_3_, 125 MHz), δ 16.0 (q, C-25), 17.4 (q, C-24), 17.5 (2 x q, C-26, C-29), 18.7 (t, C-6), 21.6 (q, C-30), 23.8 (t, C-11), 26.5 (t, C-16), 28.5 (t, C-2), 28.7 (t, C-15), 28.8 (q, C-23), 31.0 (t, C-21), 33.3 (t, C-7), 37.2 (t, C-22), 37.3 (s, C-10), 38.8 (t, C-1), 39.4 (d, C-20), 39.6 (d, C-19), 39.9 (s, C-4), 40.3 (s, C-8), 42.4 (s, C-14), 48.0 (d, C-9), 48.1 (s, C-17), 53.2 (d, C-18), 55.7 (d, C-5), 83.3 (d, C-3), 125.5 (d, C-12), 140.1 (s, C-13), 171.3 (s, C-28), 51.7 (q, OMe), *p*-NO_2_Bz [123.9 (2 × d), 131.0 (2 × d), 134.9 (s), 150.8 (s), 163.8 (s)]. ESIMS m/z 642 [M + Na]^+^ (100). HRESIMS m/z 642.3779 [M + Na]^+^ (calculated for C_38_H_53_NO_6_, 642.3771).

### 4.2. In Vitro Drug Sensitivity Assay

#### 4.2.1. *Acanthamoeba* strains 

The anti-*Acanthamoeba* activity of molecules was evaluated against the *Acanthamoeba castellanii* Neff (ATCC 30010) type strain from the American Type Culture Collection and *Acanthamoeba griffini* obtained in previous studies [13,44]. Those strains were grown axenically in Peptone Yeast Glucose (PYG) medium (0.75% (w/v) proteose peptone, 0.75% (w/v) yeast extract, and 1.5% (w/v) glucose) containing 40 μg of gentamicin ml−1 (Biochrom AG, Cultek, Granollers, Barcelona, Spain).

#### 4.2.2. In Vitro Effect Against the Trophozoite Stage of *Acanthamoeba* spp.

The anti-*Acanthamoeba* activities of the compounds were determined by the Alamar Blue assay as previously described [13,45]. Briefly, *Acanthamoeba* strains were seeded in duplicate on a 96-well microtiter plate with 50 μL from a stock solution of 10^4^ cells mL^−1^. Amoebae were allowed to adhere for 15 min and 50 μL of serial dilution series of the eye drop solution was added. Finally, the Alamar Blue assay reagent (Bioresource, Europe, Nivelles, Belgium) was added into each well at an amount equal to 10% of the medium volume. The plates were then incubated for 96 h at 28 °C with a slight agitation and the emitted fluorescence was measured with an Enspire microplate reader (PerkinElmer, Massachusetts, USA) at 570/585 nm.

#### 4.2.3. In Vitro Effect Against the Cyst Stage of *Acanthamoeba* spp.

The cysticidal activity was determined by the Alamar Blue assay at 144 h and confirmed visually by inverted microscopy. Cysts of both strains were prepared as described by Lorenzo-Morales et al. (2008) [46]. Briefly, trophozoite were transferred from PYG medium based cultures (trophozoite medium) to Neff´s encystment medium (NEM; 0.1 M KCl, 8 mM MgSO_4_·7H_2_O, 0.4 mM CaCl_2_·2H_2_O, 1 mM NaHCO_3_, and 20 mM ammediol (2-amino-2-methyl-1,3-propanediol; Sigma Aldrich Chemistry Ltd., Madrid, Spain), pH 8.8, at 25 °C) and were cultured in this medium with gentle shaking for a week in order to obtain mature cysts. After that, mature cysts were harvested and washed twice using the PYG medium.

A serial dilution of the tested molecules was made in PYG. The in vitro susceptibility assay was performed in sterile 96-well microtiter plates (Corning™). To these wells the drug concentration to be tested and 5 × 10^4^ mature cysts of *Acanthamoeba*/ml were added. The final volume was 100 μL in each well. After 7 days of incubation with the drugs, the plate was centrifuged at 3000 rpm for 10 min. The supernatant was removed and replaced with 100 µL of fresh medium PYG in each well. Finally, 10 μL of the Alamar Blue assay reagent (Biosource, Europe, Nivelles, Belgium) was placed into each well, and the plates were then incubated for 144 h at 28 °C with slight agitation and the emitted fluorescence was periodically examined with an Enspire microplate reader (PerkinElmer, Waltham, MA, USA) at 570/585 nm.

#### 4.2.4. Cytotoxicity Activity

Cytotoxicity of the selected compounds was evaluated after 24 h incubation of murine macrophage J774.A1 cell line (ATCC # TIB-67) with different concentrations of the tested compounds at 37 °C in a 5% CO_2_ humidified incubator. The viability of the macrophages was determined with the Alamar Blue assay as previously described [47]. 

#### 4.2.5. Double-Stain Assay for Programmed Cell Death Determination

A double-stain apoptosis detection kit (Hoechst 33342/PI; GenScript, Piscataway, NJ, USA) and an inverted confocal microscope (Leica DMI 4000B) were used. The experiment was carried out by following the manufacturer’s recommendations, and 10^5^ cells/well were incubated in a 24-well plate for 24 h with the previously calculated IC_90_. The double-staining pattern allows the identification of three groups in a cellular population: Live cells will show only a low level of fluorescence, cells undergoing PCD will show a higher level of blue fluorescence (as chromatin condenses), and dead cells will show low-blue and high-red fluorescence (as the propidium iodide stain enters the nucleus).

#### 4.2.6. Intracellular ROS Production Using CellROX® Deep Red Staining 

The generation of intracellular ROS was detected using the CellROX® Deep Red fluorescent probe (Invitrogen). The cells were treated with IC90 of both 5 and 7 for 24 h and exposed to CellROX® Deep Red (5 μM, 30 min) at 26 °C in the dark. Cells were observed in a Leica TSC SPE-confocal microscope equipped with inverted optics at λexc = 633 and λem = 519 nm.

#### 4.2.7. Analysis of Mitochondrial Membrane Potential

The collapse of an electrochemical gradient across the mitochondrial membrane during apoptosis was detected with the JC-1 mitochondrial membrane potential detection kit (Cell Technology). After being treated with IC_90_ of the test solution for 24 h, the cells were centrifuged (1000 r.p.m. × 10 min) and resuspended in JC-1 buffer. After that, 100 µL of each treated culture was added to a black 96 well plate (PerkinElmer) then 10 µL of JC-1 was added and incubated at 26 °C for 30 min. Analysis for the mean green and red fluorescence intensity was done using an Enspire microplate reader (PerkinElmer, Waltham, MA, USA) after 30 minutes. As well as images were taken on an EVOS fluorescence microscope from the Advanced Microscopy Group (AMG). The staining pattern allows the identification of two groups in a cellular population: Live cells will show only red fluorescence; and cells with low mitochondrial potential, (undergoing PCD) will show a higher level of green and red fluorescence.

#### 4.2.8. Measurement of ATP

The ATP level was measured using a CellTiter-Glo Luminescent Cell Viability Assay. The effect of the drug on the ATP production was evaluated by incubating (10^5^) of cells/ml with the previously calculated IC_90_ of the selected derivatives.

#### 4.2.9. Statistical Analysis 

All data are expressed as mean ± SD. For statistical comparisons between two groups, multiple comparisons were performed by a two factor analysis of variance (ANOVA), and a *p*-value (*p*) < 0.05 denoted the presence of a statistically significant difference. Statistical analyses were carried out using the GraphPad Prism8.0.2 software program (GraphPad Software, San Diego, CA, USA).

## 5. Conclusions

All tested derivatives demonstrated activity against *Acanthamoeba* spp. Based on the activity and cytotoxicity results compounds 5 and 7 were the most potent drug; higher activity versus lower toxicity. The structure-activity relationship (SAR) analysis showed that a substituent at C-3 or C-28 on the core skeleton of ursolic acid was relevant for the activity. In addition, both derivatives possessed good cysticidal activity against both tested strains. Those products could induce PCD in the parasite by the mitochondrial pathway. Chemical modification of triterpenoids as ursolic acid could be considered as a potent route to design new and effective amoebicidal drugs.

## Figures and Tables

**Table 1:** The 50% inhibitory concentrations (IC_50s_)_,_ the cytotoxic concentrations 50 (CC_50_) in µM and the selectivity index (SI) values for the compounds used in this study against *Acanthamoeba* spp. and murine macrophages measured using the Alamar blue assay. Mean concentration ± SD.

Compounds	*A. Castellanii* Neff IC_50_	*A. Griffini* IC_50_	Murine Macrophages CC_50_	SI for *A*. Neff	SI for *A. Griffini*
1	40.0 ± 2.7	15.2 ± 0.3	17.7 ± 3.3	0.4	1.2
2	24.2 ± 0.3	11.1 ± 0.8	93.2 ± 7.2	3.9	8.4
3	34.9 ± 1.5	10.2 ± 0.5	>100	>2.9	>9.7
4	21.9 ± 0.9	8.7 ± 0.4	26.3 ± 1.7	1.2	3.0
5	22.7 ± 1.4	6.9 ± 1.5	>100	>4.4	>14.5
6	37.1 ± 1.3	9.1 ± 1.0	>100	>2.7	>11.0
7	21.4 ± 1.3	6.7 ± 1.8	>100	>4.7	>14.9
8	33.5 ± 2.1	11.5 ± 0.8	>100	>3.0	>8.7
Chlorhexidine*	3.02 ± 0.89	3.73 ± 0.98	7.40 ± 0.39	2.5	2.0

* Reference compound.
